# Increased scene complexity during free visual exploration reveals residual unilateral neglect in recovered stroke patients

**DOI:** 10.1016/j.neuropsychologia.2022.108400

**Published:** 2022-12-15

**Authors:** Kira Knoppe, Nadine Schlichting, Tobias Schmidt-Wilcke, Eckart Zimmermann

**Affiliations:** a Institute for Experimental Psychology, Heinrich Heine University Düsseldorf, 40225, Düsseldorf, Germany; b Neurocenter, District Hospital Mainkofen, Mainkofen A 3, 94469, Deggendorf, Germany; c St Mauritius Therapieklinik, 40670, Meerbusch, Germany; d Institute for Clinical Neuroscience and Medical Psychology, Heinrich Heine University, 40225, Düsseldorf, Germany

**Keywords:** Subacute/chronic stroke, Unilateral neglect, Eye tracking, Virtual reality, Visual attention, Perceptual load

## Abstract

Unilateral neglect is a common cognitive syndrome after stroke, which is defined as a spatially specific unawareness of the contralesional space. The syndrome is caused by disruptions of attentional networks in the brain, which impair the patients’ ability to direct attention towards the contralesional space. During recovery, patients often learn to compensate by voluntarily directing their attention to the neglected side at the expense of cognitive resources. In this study, we examined the impact of the complexity of visual input on free visual exploration behavior of unilateral neglect and apparently recovered patients. We asked whether increasing scene complexity would allow the detection of residual unilateral neglect in recovered patients by increasing the amount of cognitive resources needed for visual processing and limiting capacities for compensation. Using virtual reality, we analyzed the spatial distribution of gaze of unilateral neglect patients, patients who had, according to conventional diagnostics, recovered from the syndrome, stroke patients with no history of unilateral neglect, and age-matched healthy controls. We manipulated the complexity of an immersive virtual scene presented on head mounted displays. We identified the orientation bias towards the ipsilesional side as a sensitive and specific marker of unilateral neglect, which was present in unilateral neglect and recovered patients but absent in stroke patients with no history of unilateral neglect and controls. Increasing scene complexity exacerbated the orientation shift in unilateral neglect patients and revealed that three out of nine (33%) recovered patients had a high probability of suffering from residual unilateral neglect as estimated by a generalized linear model using the median horizontal gaze position as a predictor.

## Highlights

• Orientation bias is a sensitive and specific marker of unilateral neglect.
• Increasing scene complexity exacerbates orientation bias in unilateral neglect.
• Asymmetrical deployment of spatial attention in recovered patients.
• Increasing scene complexity during free exploration reveals residual symptoms.

## 1 Introduction

Unilateral neglect is a debilitating cognitive syndrome occurring after unilateral brain damage, defined as a spatially specific unawareness of the contralesional side of space (Driver and Vuilleumier, 2001). The exact pathomechanisms of the syndrome remain to be fully elucidated, but most theories agree that unilateral neglect is caused by disruptions of attentional networks in the brain (Gammeri et al., 2020).

In clinical practice, unilateral neglect diagnostics is most commonly based on paper-pencil-tests, such as cancellation, search or line bisection tasks (Albert, 1973; Wilson et al., 1987). There are different diagnostic criteria which can be applied to evaluate test performance, such as measures for the asymmetry of correctly detected targets along the horizontal axis with more hits on the ipsi- and more misses on the contralesional side (Wilson et al., 1987), the Center of Cancellation (CoC) (Rorden and Karnath, 2010), the % deviation from the midline in the Line Bisection Test (Schenkenberg et al., 1980) or early orientation (Pflugshaupt et al., 2004) among others. The tests require little resources and are easy to administer, but have been shown to vary substantially in their sensitivity (Azouvi et al., 2002).

Several factors have been shown to modulate unilateral neglect patients’ performance in search and cancellation tasks and to increase their diagnostic sensitivity. The manipulation of visual content by reducing stimulus saliency (Aglioti et al., 1997), increasing the total number of targets (Ten Brink et al., 2020), using dynamic backgrounds with moving distractors (Deouell et al., 2005) or increasing the complexity of a visual scene (Ogourtsova et al., 2018) significantly exacerbates neglect of and detection time for contralesional targets. Moreover, unilateral neglect has been shown to be more pronounced in dual-than in single-tasks (Blini et al., 2016; Bonato et al. 2010, 2013; Villarreal et al., 2021) and when concurrent tasks require cognitive control (Mennemeier et al., 2004; Sarri et al., 2009). Thus, when the perceptual or attentional demands of a search or cancellation task increase, spatial attention deficits become more pronounced and easier to detect.

This close link between task demands and awareness of the contralesional space reflects the patients’ ability to compensate for their deficits (Bonato, 2012) and is based on top-down modulation of sensory processing in the primary visual cortex (Vuilleumier et al., 2008). When task demands are low, patients can use their limited cognitive resources to compensate for their deficits by implementing acquired strategies. This might be particularly relevant in search tasks measuring voluntary behavior, which is driven by goals and modulated by task instructions (Karnath and Niemeier, 2002; Paladini et al., 2019). Presumably, when a task requires the use of search strategies, exploratory behavior is top-down controlled and more prone to the influence of compensatory strategies. In line with this argument, it has been shown that goal-directed search behavior is less affected by unilateral neglect than stimulus-driven reactive behavior (Karnath, 2015) and that cancellation tasks are less sensitive for unilateral neglect than the analysis of free visual exploration behavior (Delazer et al., 2018; Kaufmann et al., 2020).

The ability to visually explore and become aware of the surrounding space is a fundamental requirement for all kinds of everyday behaviors. Accordingly, spatial attention deficits measured during free visual exploration sensitively predict impairments in activities of daily living (Kaufmann et al., 2020).

In the present study, we examined how increasing the load and complexity of a virtual environment would affect free visual exploration behavior in stroke patients with and without unilateral neglect. To this end, we analyzed eye movements during free visual exploration of an immersive virtual scene displayed on a head-mounted display. We manipulated scene complexity by adding objects, sounds and movements to a desert landscape.

We predicted that stroke patients who never experienced unilateral neglect symptoms as well as age-matched healthy controls would explore both sides of space equally. Unilateral neglect patients, classified by conventional unilateral neglect tests, were expected to show a pronounced orientation bias towards their ipsilesional and a consequent neglect of their contralesional space irrespective of scene complexity. Given the low sensitivity of conventional unilateral neglect tests (Azouvi et al., 2002; Delazer et al., 2018; Kaufmann et al., 2020), we assumed that all patients classified as suffering from unilateral neglect by conventional tests were severely impaired and unable to compensate for their deficits even when scene complexity was low. However, in a subsample of apparently recovered patients, who were diagnosed with unilateral neglect at some point after their stroke but did not show acute signs of unilateral neglect in the conventional tests, we expected to find a clear impact of scene complexity on the free visual exploration pattern. In the low complexity scene, recovered patients were expected to engage acquired compensatory exploration strategies, which would allow them to explore both sides of space equally. With the increase of scene complexity, we predicted to find the characteristic orientation bias towards the ipsilesional side and neglect of the contralesional space as the limited cognitive resources needed for compensatory strategies were already needed for processing the visual input.

## 2 Materials and methods

### 2.1 Participants

Overall, we screened 51 stroke patients after ischaemic or haemorrhagic stroke in the territory of the middle cerebral artery (MCA). We included both right- and left-sided stroke patients, but excluded both-sided strokes. Patients were included when they were between 18 and 90 years of age and had normal or corrected-to-normal vision. Exclusion criteria were the presence of severe cognitive, psychiatric or neurodegenerative disorders and visual field defects. All participants gave their informed written consent before study enrolment. The study was approved by the ethics committee of the medical faculty of the Heinrich-Heine-University Duesseldorf in accordance with the declaration of Helsinki (2019–614).

Four patients were excluded because of visual field defects. One patient was excluded because they showed left-sided unilateral neglect in the Star Cancellation Test (22/27) and right-sided neglect in the Line Crossing Test (18/15).

The final sample consisted of 46 patients. 29 patients had no history of unilateral neglect (15 right-sided strokes; f = 6; mean age = 74 ± 10 years; mean duration since stroke = 27 ± 10 days), 9 patients (7 right-sided strokes; f = 4; mean age = 77 ± 8 years; mean duration since stroke = 38 ± 33 days) experienced unilateral neglect in the acute stage after stroke but did not show symptoms upon study admission in conventional tests (further referred to as “recovered”), 8 patients (only right-sided strokes; f = 4; mean age = 80 ± 8 years; mean duration since stroke = 28 ± 7 days) showed severe unilateral neglect both during clinical examination and neuropsychological testing. One patient in this group (patient 43; ischaemic stroke in right MCA-territory 26 days prior to VR session) was not able to complete neuropsychological testing because of health issues on the day of testing, but suffered from severe unilateral neglect symptoms as confirmed by her treating neuropsychologist and was, thus, assigned to the unilateral neglect group. The results of the stroke patients were compared to a group of 11 neurologically healthy, age-matched controls (f = 4; mean age = 67 ± 23 years).

### 2.2 Unilateral neglect assessment

Unilateral neglect was assessed with two cancellation tests in paper-pen-format (Line Crossing (Albert, 1973) and Star Cancellation (Wilson et al., 1987), a modified Baking Tray Task (Tham, 1996) and a reading task. The Line Crossing (Albert, 1973) and Star Cancellation tests (Wilson et al., 1987) were printed on a horizontally oriented 21 cm × 29.7 cm sheet of paper and placed centrally in front of the participants.

In the Line Crossing Test (Albert, 1973), participants were instructed to cross out all the lines they could find on the paper (40 lines of 2.5 cm in 6 different orientations distributed evenly on the sheet). The number of omitted lines was counted. If at least 70% of omitted lines were on the same side, unilateral neglect was diagnosed.

In the Star Cancellation test (Wilson et al., 1987), the targets were 56 small stars, which were evenly distributed on the sheet. 52 larger stars, 13 letters and 10 words served as distractors. The number of small stars crossed on each side was counted. If at least 70% of the omitted stars were on the same side, unilateral neglect was diagnosed.

The modified Baking Tray Task (Tham, 1996) consisted of 12 wooden cubes the participants were asked to evenly distribute on a tray “as if they were buns”. The number of cubes per side was counted. Unilateral neglect was diagnosed when at least 8 out of 12 cubes are placed on the patient's ipsilesional side.

The reading task consisted of five 21 cm × 29.7 cm sheets of paper on which six letters were printed horizontally. The participants were asked to read the letters aloud. The total number of letters correctly read was counted. Any deviation from perfect (30) was considered a sign of unilateral neglect, if the omitted letters were located on the contralesional side of the sheet. All patients who omitted letters only did so on their contralesional side.

There was no time limit on any of the tests. Visual field defects were clinically assessed moving a pen from the left or right into the patient's visual field towards the centre. Patients were asked to tell the examiner when they detected the pen. When the pen was only detected in the patient's visual field's centre, a complete homonymous hemianopia was diagnosed. This procedure was repeated two times. Central gaze fixation was controlled by the examiner.

### 2.3 Patient classification

Stroke patients were assigned to the unilateral neglect group when their test performance in at least two of the tests met the criteria for a unilateral neglect diagnosis. Additionally, we differentiated between patients who have never been diagnosed with unilateral neglect and those who had suffered from the syndrome either in acute care or during rehabilitation as reported by their treating neuropsychologists but who did not meet the criteria for a unilateral neglect diagnosis upon testing for the study. The latter are further referred to as “recovered”.

### 2.4 Apparatus

We used an HTC VIVE Pro Eye for the experiment. The VR Hardware ran with the Steam VR version 1.14.16. The screen was dual OLED 3,5″ diagonal with a resolution of 1440 × 1600 pixels per eye (2880 × 1600 pixels combined) and a refresh rate of 90 Hz. The field of view was at 110°, which also represents the trackable field of view for eye tracking, which was integrated into the HTC VIVE Pro Eye. Tobii Eye Tracking accuracy within FOX 20 was 0.5°–1.1°. Gaze origin and gaze direction were recorded in a three-dimensional right-handed coordinate system. The experiment was programmed in Unity version 2019.1.13 fl using C# programming language. Audio was played on high-resolution headphones attached to the HTC VIVE Pro Eye headset. Two base stations were used to detect the VR headset.

The experiment was run on an ALIENWARE 15 R4 with an Intel Core i9-8950HK CPU Coffee Lake-H six-core processor at 2.9 GHz and 8192 MB NVIDIA Ge Force GTX 1070 Mobile GPU graphics adapter. The laptop contained a 32 GB (2 × 16 GB) SO-DIMM DDR4-2666 RAM physical memory and a Toshiba MQ02ABD100H 1 TB 7200 RPM hybrid HDD storage. The operating system was Microsoft Windows 10 Pro build 10.0.18362. SR_Runtime was used to enable eye tracking.

Eye tracking data were preprocessed by the Tobii XR SDK (Tobii Technology Inc. Sweden). The SDK provides a three-dimensional gaze direction vector in the right-handed coordinate system, containing normalized gaze data between −1 and +1. Time information was recorded with a timestamp in SRanipal SDK provided by HTC, which also recorded the frame sequence. Because of the richness of the scenes computational power occasionally reached its limits, causing lags in data logging (i.e., the sampling rate would drop from 90 Hz to 45 Hz or lower). This problem did not occur throughout the whole experiment, but for short moments. We removed all data that could have been affected by these performance problems from the analysis.

### 2.5 Task

The virtual environment “Tropical Forest Pack” (created by “Baldinoboy”) was purchased from the Unity Asset Store. The pack contained photo scanned rocks, modelled trees, ground material and falling leaf particles. We used four different scenes (bridges, waterfall, pond and forest), which were chosen randomly. After a five-point calibration, the session started with a desert-like environment (low complexity) and participants were told to look around to get used to the headset and the environment. After 1 min, trees, plants and bridges were added to the scene (medium complexity), but the scene remained static and did not have sound. After another minute, movement in the trees by wind, waterfalls, fire and/or steam and sounds (bird noises and the sound of wind) were added (high complexity) The sound was played equally on both sides. Every condition was presented for 1 min. We included a video of the different scenes in the supplementary material.

### 2.6 Measures of visual exploration

Analyses on visual exploration patterns were based on fixational eye movements. The pupil data outputted by the Tobii Eye Tracking system is scaled from −1 to 1 in x-, y- and z-direction (horizontal, vertical, and depth position) relative to the HMD position in space. We converted this measure to a measure of rotational degree (°), which can be interpreted in the same way as visual degree. Horizontal and vertical pupil locations were calculated as:

$$Horizontal\;eye\;position=\left(\frac{atan\left(\frac{x_{pupil}}{z_{pupil}}\right)}{\pi }*180\right)-90$$

$$Vertical\;eye\;position=\left(\frac{acos\left(\frac{y_{pupil}}{\sqrt{x_{pupil}^{2}+y_{pupil}^{2}+z_{pupil}^{2}}}\right)}{\pi }*180\right)-90$$

Based on these conversed pupil position data we calculated eye movement velocities. We classified all periods in which eye velocity remained smaller than 1.1°/s and which lasted longer than 80 ms as fixations. Fixations were further classified as ipsi- or contralesional dependent on horizontal pupil location and lesion site. Thus, for right-hemispheric stroke patients, all fixations measured on the right side were classified as ipsi- and all left-sided fixations as contralesional and vice versa for left-hemispheric stroke patients. For control patients, we classified fixations in the right visual field as ipsilesional in order to have a standardized measure for all groups. This classification did not affect the study results as controls explored both sides of space evenly.

#### 2.6.1 Number of fixations (Nfix)

The total number of fixations was calculated for each gaze direction (ipsi - and contra-lateral) and complexity condition separately.

#### 2.6.2 Laterality index (LI)

The laterality index is a measure for left-right differences or hemispheric dominance, which is commonly used in functional magnetic resonance imaging (fMRI) studies (Ito and Liew, 2016). In fMRI studies, active voxels in regions of interest are counted in both hemispheres and the ratio of left-hemispheric voxels to all voxels is calculated. Score can range between −1 and +1, indicating right- or left-hemispheric dominance, respectively.

Here, we used the same approach to calculate the ratio of contralesional fixations to all fixations made across the whole field of view in order to determine orientation biases. A score of roughly zero indicates an even distribution of fixations across both sides of space, while a negative score indicates neglect of the contralesional side and a positive score of the ipsilesional side of space. We calculated the laterality index separately for each complexity condition as follows:

$$LI=\left(N_{fix,ipsi}-N_{fix,contra}\right)\;/\;\left(N_{fix,ipsi}+N_{fix,contra}\right)\text{.}$$

#### 2.6.3 Spatial distribution of fixations

We calculated the mean and median gaze position in the horizontal pane for all groups as measures of spatial orientation. We controlled for lesion site by calculating the mean and median based on the number of ipsi- and contralesional fixations. A higher mean or median indicates orientation towards the ipsilesional side. For control patients, mean and median is the same as the mean and median horizontal gaze position, thus, a higher mean and median indicates an orientation shift towards the right. The width of the visually explored field was calculated as the interquartile range (IQR) of fixation positions on the horizontal axis.

#### 2.6.4 Visual scanning efficiency

Scan path analyses based on entropy measures provide the possibility to quantify how the visual field is explored in terms of orderliness, and has been applied in a variety of tasks and settings (for a review see Shiferaw et al., 2019). We divided the visual field into 5 × 5° bins and recorded, for each participant and in each complexity condition, transitions between these bins in a transition frequency matrix. This matrix, in a next step, was used to calculate transition probabilities and further measures. We calculated gaze transition entropy (GTE) and stationary gaze entropy (SGE) to quantify visual scanning efficiency (Krejtz et al., 2015). While GTE estimates a level of randomness in the visual scanning pattern (higher GTE-values correspond to more random scanning paths and more frequent switching), SGE provides a measure of spatial dispersion of visual attention (higher SGE-values are related to a more uniform distribution of visual attention over states) (Shiferaw et al., 2019).

### 2.7 Data analysis

#### 2.7.1 Measures of visual exploration

All above described measures were analyzed by means of mixed ANOVAs including the factors *group* (controls, unilateral neglect, recovered, and stroke without history of neglect; between-participants factor), *complexity* (low, medium, or high scene complexity; within-participants factor), and, if applicable, *direction* (ipsi- or contra-lateral; within-participants factor) using the rstatix package (Kassambara, 2021) in *R* (version 4.03, R Core Team, 2017). Greenhouse-Geisser correction was applied if the sphericity condition was not met. Significant effects were decomposed by means of post-hoc pairwise *t*-tests using Bonferroni adjusted *p*-values.

#### 2.7.2 Prediction model

Using the above described measures that showed significant differences for group complexity (median, mean, IQR, LI), we aimed to predict the presence and severity of unilateral neglect in apparently recovered patients. To this end, we developed a set of generalized linear models (GLMs) with different combinations of predictors to fit logistic regression models in *R*.

In a first step, to predict the presence of pathological unilateral neglect, we trained models on measures in the high complexity condition only. Data from apparently recovered neglect patients were left out during model training and used as the final test-sample. Model selection was based on the Akaike information criterion (AIC). If a model is more than 2 AIC units lower than another, it is considered significantly better. Evidence for the inclusion or exclusion of predictors will be reported as the difference in AIC values of the model including the predictor and the model without the predictor. Estimates of the final model will be reported as logits. The final model was validated with 1000 bootstrap replicates. During each of the 1000 iterations data from one randomly chosen patient of each group (control, stroke, neglect) was left out during model training and subsequently used to test the model's prediction performance. To further assess discrimination performance, we calculated the area under the receiver operating curve (AUC-ROC) during each iteration using the *pROC* package in *R*(Robin et al., 2011). Next, the data of apparently recovered patients was applied to the final model trained on the data of all controls, stroke and unilateral neglect patients. The outcome of this step is a probability value of being diagnosed with unilateral neglect.

We obtained one probability measure for each complexity level and for each apparently recovered patient, enabling us to assess the progression of neglect severity from low to high complexity environments. Next, for each apparently recovered patient and for each left-out patient during the bootstrapping validation, we fitted a linear regression through these three datapoints (i.e., probability of unilateral neglect in high, medium and low complexity environments) and used the slope and intercept estimates to judge severity of remaining unilateral neglect symptoms.

## 3 Results

### 3.1 Participants

Participants’ demographic characteristics are listed in the methods section (2.1. Participants). Individual test results of all patients are listed in the supplementary material. The groups did not differ with regards to their age (*H*_(4)_ = 4.37, *p =*.36). With regard to lesion site, all patients classified as unilateral neglect patients suffered from a right-sided stroke, while 2 out of 9 (22%) recovered patients and 15 out of 29 (52%) stroke patients with no history of unilateral neglect suffered from a left-sided stroke. In order to rule out that differences in spatial orientation were associated with lesion site rather than unilateral neglect, we analyzed orientation differences between left- and right-sided stroke patients with no history of unilateral neglect. Analyses of the impact of lesion site on median, mean and LI did not reveal significant differences between left- and right-sided stroke patients. Moreover, when excluding all left-sided stroke patients from groupwise analyses, the results did not change significantly. All analyses mentioned here are listed in the supplementary material.

### 3.2 Measures of visual exploration

#### 3.2.1 Number of fixations

Number of fixations for each group in each complexity condition, split for contra- and ipsilateral viewing direction are depicted in Fig. 2A. A three-way mixed ANOVA testing the effect of group, complexity and direction on the number of fixations revealed a significant interaction between group and direction (*F*_3,53_ = 11.14, *p* < .001, η^2^_*p*_ = .39). Post-hoc comparisons showed that the effect of direction was only present in neglect (*p* < .001) and recovered neglect patients (*p* < .001), who showed a pronounced preference for the ipsilesional side. Controls and stroke patients with no history of unilateral neglect distributed their fixations over both sides of space equally. Further, we found a statistically significant main effect of direction (*F*_1,53_ = 20.60, *p* < .001, η^2^_*p*_ = .28) and complexity (*F*_1.61,85.25_ = 17.90, *p* < .001, η^2^_*p*_ = .25). Post-hoc comparisons revealed that significantly more fixations were made in the high complexity than in the low complexity condition (*p <*.05). These results show that patients with unilateral neglect do not make fewer fixations than controls or stroke patients without the syndrome per se, but that they differ in the spatial distribution of fixations, making significantly more fixations on their ipsilesional side.

#### 3.2.2 Fixation duration

In a three-way mixed ANOVA including the factors group, complexity and direction we found significant main effects of direction (*F*_1,53_ = 7.56, *p* < .001, η^2^_*p*_ = .13) and complexity (*F*_1.27,67.57_ = 13.68, *p* < .001, η^2^_*p*_ = .21). Pairwise post-hoc comparisons showed that fixations were significantly longer on the ipsilesional as compared to the contralesional side (*p <*.05). Moreover, fixations in the high complexity condition were significantly longer than in the low complexity condition (*p <*.01).

We found interaction effects between group and direction (*F*_3,53_ = 3.58, *p* < .05, η^2^_*p*_ = .17), complexity and direction (*F*_1.13,59.66_ = 4.03, *p* < .05, η^2^_*p*_ = .07) and between group, complexity and direction (*F*_3.38,59.66_ = 2.56, *p* < .05, η^2^_*p*_ = .14). Decomposing the interaction effect between group and direction revealed that unilateral neglect patients made significantly longer fixations on their ipsi-than on their contralesional side (*p <*.001). Pairwise post-hoc comparisons of fixation duration within the different complexity conditions did not reveal significant differences in durations of ipsi- and contralesional fixations when considering all groups (both *ps >*.05).

#### 3.2.3 Laterality index

In a two-way mixed ANOVA including the factors group and complexity we found a main effect of group (*F*_3,53_ = 10.89, *p* < .001, η^2^_*p*_ = .38). Pairwise post-hoc comparisons showed that unilateral neglect patients had significantly higher LI-scores than all other groups (all *ps <*.05), meaning that they showed a significantly higher imbalance of fixations with more fixations on the ipsilesional than on the contralesional side. LI-scores of apparently recovered patients were significantly lower than scores of unilateral neglect patients (*p <*.05), but significantly higher than the ones measured in controls and stroke patients with no history of unilateral neglect (both *ps <*.01). This shows that apparently recovered patients also showed a significant imbalance in the spatial distribution of their fixations with more fixations on their ipsilesional side, which was less extreme than the one measured in unilateral neglect patients. Stroke patients with no history of unilateral neglect and controls had laterality indices of roughly zero, indicating an even distribution of fixations across both sides of space (see Fig. 3).

#### 3.2.4 Spatial distribution of fixations

A potential orientation bias was analyzed using the mean and median gaze position on the horizontal axis in relation to the lesion site, further simply referred to as mean and median (see method section, 2.6.3.).

A mixed ANOVA testing the effects of groups and complexity on mean revealed main effects of group (*F*_3,53_ = 16.01, *p <*.001, η^2^_p_ = .48), complexity (*F*_1.67,88.35_ = 5.30, *p <*.01, η^2^_p_ = .09) and an interaction between group and complexity (*F*_5,88.35_ = 2.98, *p <*.05, η^2^_p_ = .14). Pairwise post-hoc comparisons revealed that the group of unilateral neglect had a significantly higher mean than all other groups (all *ps <*.001), indicating a stronger orientation shift towards the ipsilesional side. Recovered patients also oriented significantly more towards their ipsilesional side than stroke patients with no history of unilateral neglect and healthy controls (both *ps <*.05), but their orientation bias was significantly less extreme than the one measured in unilateral neglect patients (*p <*.001). Analyzing the effect of scene complexity did not reveal a significant difference between the conditions (both *ps >*.05). Decomposing the interaction effect of group and complexity also did not reveal a statistically significant impact of scene complexity on the orientation bias in the different groups (see Fig. 4A).

A mixed ANOVA testing the effects of groups and complexity on median revealed main effects of group (*F*_3,53_ = 15.21, *p <*.001, η^2^_p_ = .46), complexity (*F*_1.79,94.76_ = 6.56, *p <*.01, η^2^_p_ = .11) and an interaction between group and complexity (*F*_5.36,94.76_ = 3.62, *p <*.01, η^2^_p_ = .17). Pairwise post-hoc comparisons showed that unilateral neglect patients oriented significantly more towards their ipsilesional side than all other groups (all *ps <*.001). The median in the recovered group was significantly higher than in controls and stroke patients without a history of unilateral neglect (*p <*.01) and significantly lower than in the unilateral neglect group (*p <*.001), showing again that recovered patients oriented significantly more towards their ipsilesional side than controls and stroke patients with no history of the disorder, but that their orientation bias was significantly less extreme than the one measured in unilateral neglect patients. Pairwise comparisons of the different scene complexities did not reveal significant differences (*ps >*.5). Decomposing the interaction effect between group and complexity showed that the orientation bias in unilateral neglect patients became more extreme when scene complexity increased (low vs. medium and high complexity both *ps <*.05) (see Fig. 4B).

A mixed ANOVA testing the IQR in the horizontal view pane revealed main effects of group (*F*_3,53_ = 7.28, *p* < .001, η^2^_*p*_ = .29) and complexity (*F*_2,106_ = 6.74, *p* < .01, η^2^_*p*_ = .11). Pairwise post-hoc group comparisons showed that healthy controls had significantly higher IQRs than all stroke patients with and without unilateral neglect, meaning that the space they explored was significantly wider (all *ps <*.01). Analyzing the effect of scene complexity revealed that the visually explored space was significantly wider in the medium and high than in the low complexity condition (both *ps <*.01). However, this effect was only measured in the control group and in stroke patients with no history of unilateral neglect, who widened their field of exploration when objects were added to the scene (low vs. medium scene complexity in stroke and control group both *ps <*.01).

The presented results on the spatial distribution of fixations revealed that both unilateral neglect patients and those who recovered from the syndrome show a pronounced orientation bias towards their ipsilesional side as reflected in the mean and median horizontal gaze position in relation to their lesion site. Additionally, we found that the width of the visually explored field was significantly smaller in all stroke patients independently from unilateral neglect as reflected in the IQR of horizontal gaze positions. However, stroke patients with no history of unilateral neglect widened their field of exploration when scene complexity increased, an effect that was found in healthy controls as well, but not in unilateral neglect or recovered patients.

#### 3.2.5 Visual scanning efficiency

Mixed ANOVAs testing the effects of group and complexity on stationary gaze entropy and transition entropy did not reveal significant differences between the groups (*ps >*.4). We found a significant main effect of complexity on stationary gaze entropy (*F*_1.46,67.05_ = 4.26, *p* < .05, η^2^_*p*_ = .09). Pairwise post-hoc comparisons showed that the stationary gaze entropy was higher in the medium and high than in the low complexity condition (both *ps <*.05). Thus, when scene complexity increased, visual attention was more uniformly distributed. Given the lack of any group effects, patients and control participants do not differ in their spatial dispersion of visual attention and how orderly they scan their visual environment (see Fig. 5).

### 3.3 Prediction model

The prediction model included only the *mean horizontal gaze position* (*mean*; slightly favored over *median*, ΔAIC = −2.29, and *LI*, ΔAIC = −0.78). *IQR* and interaction terms did not improve the model fit.

Based on the mean horizontal gaze position, we were able to discriminate between the group of unilateral neglect patients on one side and stroke patients with no history of unilateral neglect and healthy controls on the other (see Fig. 6A, left column). As shown in Fig. 6B, the estimated probability of a “unilateral neglect” diagnosis increased when the mean horizontal gaze position was shifted to the ipsilesional side (the probability of a “unilateral neglect” diagnosis was above 50% when mean > 40°). Across all complexity conditions, the good discrimination performance of the model was also reflected in the ROC analyses, in which high AUC values were obtained in all three complexity conditions (Fig. 6A, right column; AUC_low_ = 0.87, AUC_medium_ = 0.97, AUC_high_ = 0.9).

When applying the model on the group of recovered patients, we found that one patient had a high probability of unilateral neglect (*p*_neglect_ = 76.80%) in the medium, but not in the high complexity condition (*p*_neglect_ = 6.94%). Two other patients had a high probability of a ‘unilateral neglect’ diagnosis in the high complexity (*p*_neglect_ = 86.80% and 60.5%), but not in the medium complexity condition (*p*_neglect_ = 35.90% and 31.30%, respectively).

Fig. 7 shows the data for two sample participants in the recovered group, one of which shows lateralized fixation patterns with increasing scene complexity. The prediction model was able to capture this increase in pathological behavior. The other sample participant did not show a change in gaze behavior with increasing scene complexity, captured by the prediction model with low predicted ‘unilateral neglect’ diagnosis.

Taken together, our prediction model successfully identified pathological behavior in three out nine (33%) apparently recovered neglect patients based on the behavioral dynamics caused by increased scene complexity.

## 4 Discussion

In the present study, we examined the impact of scene complexity on free visual exploration behavior of unilateral neglect patients in comparison to stroke patients with and without a history of the disorder and age-matched healthy controls. We aimed to investigate whether increasing scene complexity would result in a more pronounced neglect of the contralesional space and a higher sensitivity for spatial awareness deficits.

For this, we first sought to identify a sensitive and specific marker of unilateral neglect, that would robustly discriminate between pathological and healthy free visual exploration behavior. The analysis of different eye movement parameters showed that the groups did not differ with regards to neither the total number of fixations, nor the overall fixation duration nor to how orderly they scanned the visual scene as measured by stationary and transition gaze entropy. Across all groups, participants made more and longer fixations and scanned the scene more uniformly when scene complexity increased. Given the lack of interaction effects between group and complexity, we conclude that these effects were independent from unilateral neglect.

As expected, the characteristic abnormalities in free visual exploration behavior of unilateral neglect patients were spatial in nature, affecting the exploration of the contralesional space. In contrast to healthy controls and stroke patients with no history of the disorder, who explored both sides of space, unilateral neglect patients showed a significant orientation shift towards their ipsilesional and consequent neglect of their contralesional space. This ipsilesional orientation shift could be measured as an index of fixation asymmetries or as the mean and median fixation position on the horizontal axis.

This is in line with previous studies, which analyzed eye movement behavior in unilateral neglect using stationary eye tracking devices and either search tasks (Husain et al., 2001; Niemeier and Karnath, 2003), exploration of naturalistic photographs (Kaufmann et al., 2020; Muri et al., 2009; Paladini et al., 2019) or a combination of both (Delazer et al., 2018; Machner et al., 2018). Independently from the method of choice, the orientation bias towards the ipsilesional side was consistently identified as a correlate of unilateral neglect. The orientation shift could be measured and expressed by the relative time participants spent exploring their ipsi-versus their contralesional side (Johnston and Diller, 1986), the number of fixations measured on the ipsi-versus the contralesional side (Cazzoli, 2015, LLorens and Noé, 2016; Machner et al., 2012) and by the mean and median fixation position on the horizontal axis (Fruhmann Berger et al., 2008; Kaufmann et al., 2020).

Importantly, the ipsilesional orientation shift was found in all but one patient who were classified as suffering from unilateral neglect by conventional tests across all complexity conditions. Thus, our results are in line with our hypothesis that severely affected unilateral neglect patients would neglect their contralesional side independently from the complexity of the virtual scene. Only one patient explored both sides of space in the low and medium complexity condition and showed the orientation shift only in the high complexity condition. This patient differed from the rest of the unilateral neglect group with regards to their performance in conventional tests, too. While all other patients in the group showed severe unilateral neglect in both cancellation tests, this patient only neglected their contralesional side of space in the baking tray and the reading task. Thus, it is possible that the conventional tests used in this study measure closely related but distinct constructs, which might affect free visual exploration behavior differently. Given that all other patients in the unilateral neglect group were classified as suffering from the syndrome based on the cancellation tests and showed pronounced neglect of their contralesional side across all complexity conditions, it seems that free visual exploration behavior is most closely related to search behavior as assessed in cancellation tasks. This is in line with the study by Kaufmann et al. (2020) who were able to detect 93.75% of neglect patients using eye movement analysis as compared to the Star Cancellation Test (Wilson et al., 1987). Future studies with larger sample sizes and a collection of different neuropsychological tests are needed to analyze how the different tests correlate with each other and with free visual exploration behavior and what underlying factors might explain differences between the tests.

Two previous studies compared free visual exploration behavior during exploration of naturalistic photographs (Kaufmann et al., 2020) and an immersive virtual environment (Hougaard et al., 2021) with impairments in activities of daily living (ADLs). They found that 56% (Hougaard et al., 2021) and 85% (Kaufmann et al., 2020) of the patients classified as unilateral neglect patients based on systematic observations of real-life situations (Azouvi et al., 2003; Chen et al., 2012) showed the characteristic ipsilesional orientation bias during free visual exploration, respectively. A closer look into the individual data sets in the first study (Hougaard et al., 2021) reveals that patients with mild impairments in ADLs (Chen et al., 2012) did not show a pronounced ipsilesional orientation bias, but nine out of eleven (82%) moderately impaired patients and the only severely impaired patient did, which is line with the results by Kaufmann et al. (2020) and the present study. The assessments used to detect spatial impairments in ADLs (Azouvi et al., 2003; Chen et al., 2012) use the observation of spontaneous, self-initiated behaviors needed in daily life. These behaviors do not only require the ability to visually explore and become aware of the surrounding visual space, but also the ability to perform movements, interact with objects and be aware of one's own body. Thus, even though we assume that spatial awareness is a key requirement for all kinds of behaviors, ADLs require much more than that. Therefore, we suggest that the inability to visually explore one side of space and become aware of it necessarily leads to impairments in most ADLs, but that impairments in ADLs can have many different causes. However, we did not include measures for impairments in ADLs in our study, thus, we cannot make any conclusive statements about the relevance of impairments in free visual exploration behavior on real-life situations.

While a few studies only compared unilateral neglect patients with healthy controls (Husain et al., 2001; Muri et al., 2009), the comparison between stroke patients with and without unilateral neglect provided conflicting results. In some studies, the ipsilesional orientation bias was specific to unilateral neglect (Fruhmann Berger et al., 2008; Niemeier and Karnath, 2003), while in others it was found in all right-sided stroke patients independently from the disorder (Delazer et al., 2018; Machner et al., 2018). In the present study, stroke patients with no history of unilateral neglect did not differ from healthy controls, which confirms that the orientation bias is, in fact, specific to unilateral neglect and not a common symptom after stroke. We argue that the difference in the study results is due to the criteria according to which patients were assigned to the groups. In both of the abovementioned studies (Delazer et al., 2018; Machner et al., 2018), patients were classified based solely on their performance in conventional tests. These tests have been shown to vary substantially in their diagnostic sensitivity (Azouvi et al., 2002), which can lead to a considerable rate of false negatives. Thus, it is possible that patients who scored within the healthy range in conventional tests still suffered from unilateral neglect and were mistakenly assigned to the group of stroke patients with no unilateral neglect.

In the present study, we differentiated between patients who never experienced spatial awareness deficits since stroke and those who apparently recovered from them. With this setup, we aimed to examine whether some recovered patients would show the characteristic ipsilesional orientation bias during free exploration. We hypothesized that over the course of rehabilitation, some patients acquire strategies to compensate for their spatial attention deficits. We further argued that these compensatory strategies would be more readily available when behavior was guided by behavioral goals. Task instructions have been proven to significantly impact visual exploration behavior in both healthy individuals (Neider and Zelinsky, 2006) and in unilateral neglect patients (Karnath and Niemeier, 2002). For example, when individuals are instructed to search for a specific target, their search behavior is strongly influenced by their expectations of the target's location. When no instructions are given, behavior is less controlled and, thus, less prone to confounding influences of compensatory mechanisms. In line with this argument, it has already been shown that eye movement patterns during free exploration reveal unilateral neglect more sensitively than search or cancellation tasks (Delazer et al., 2018; Kaufmann et al., 2020). Importantly, the orientation bias towards the ipsilesional side correlates strongly with impairments in activities of daily living (Hougaard et al., 2021; Kaufmann et al., 2020; Machner et al., 2018), proving the clinical value of using eye movement analysis during free exploration as a diagnostic tool.

We found a significant difference between stroke patients who never experienced unilateral neglect symptoms and those who recovered from them. While stroke patients with no history of unilateral neglect evenly explored both sides of their visual field, apparently recovered patients made significantly more fixations on their ipsi-than on their contralesional side. This is in line with our hypothesis that some of the apparently recovered patients still suffered from residual unilateral neglect. Future studies should examine in how far these residual symptoms still affect the patients’ physical and cognitive functioning and independence. We are aware that our classification was also based on conventional tests, thus, it remains possible that some of the patients, who were classified as “stroke patients without a history of unilateral neglect” also suffered from unilateral neglect either before or during the study. This might especially concern patients with milder symptoms or patients with personal neglect (Caggiano and Jehkonen, 2018). Thus, we recommend the use of assessments of ADLs (Azouvi et al., 2003) in future studies as additional reference measures.

The main question of this study was how increasing the complexity of a visual scene would affect free visual exploration behavior and unilateral neglect in the different groups. As predicted, we found that unilateral neglect patients showed a pronounced ipsilesional orientation bias across all complexity conditions, which further exacerbated with the increase in scene complexity. Healthy controls and stroke patients with no history of unilateral neglect, on the other hand, evenly explored both sides of space, irrespective of the complexity of the visual scene. Thus, the results show that free visual exploration behavior in individuals without spatial awareness deficits is not affected by the complexity of the scene.

We further examined whether some of the recovered patients would be able to explore the whole width of their visual field in the low complexity condition by using acquired compensatory strategies and show an orientation bias in the high complexity condition when their attentional resources were consumed by processing the visual content. This research question is based on the finding that higher perceptual or attentional demands of a task exacerbate unilateral neglect (Blini et al., 2016; Bonato et al. 2010, 2013; Deouell et al., 2005; List et al., 2008; Ogourtsova et al., 2018). According to the “load-theory” (Lavie, 1995, 2005; Lavie et al., 2014), humans have a limited capacity to process sensory information and to control what kind of information is processed. Thus, when confronted with low attentional or perceptual demands, all information is processed, irrespective of its relevance. Moreover, the availability of free processing resources allows the individual to exert some control over the processed information and prioritize tasks by actively engaging top-down controlled strategies (Bonato, 2012). Applied to the present study, this could mean that under low perceptual load, patients who have already partially recovered from unilateral neglect and acquired compensatory strategies would be able to actively compensate for their deficit. The question on how this compensation works on a neurophysiological level would exceed the scope of this study, but the answer might lie in top-down controlled activation of visual – or even multisensory - processing areas in the damaged hemisphere (Vuilleumier et al., 2008).

**Fig. 1:**
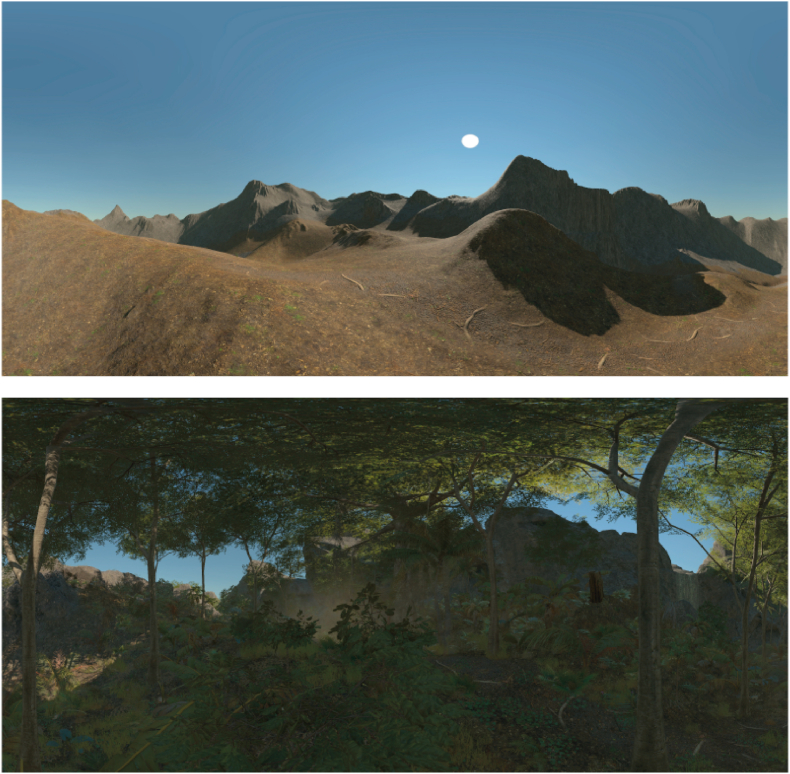
Depiction of the virtual environment in the low complexity (top) and medium complexity condition (bottom). The high complexity scene included animated objects (i.e., leaves moved by the wind, a waterfall) of the medium complexity scene. Example videos of different scenarios and complexity conditions can be found in the supplementary materials.

The load theory (Lavie, 1995, 2005; Lavie et al., 2014) further suggests that tasks with a high attentional or perceptual load – either manipulated by task difficulty (Blini et al., 2016; Bonato et al. 2010, 2013) or scene complexity (Ogourtsova et al., 2018) – consume all available processing resources, which results in processing of only the relevant task at hand (and which also explains the phenomenon of *inattentional blindness* (Mack and Rock, 1998)) and which hampers the individual's ability to exert cognitive control. The results of the present study are in line with these predictions as the characteristic ipsilesional orientation bias in the recovered group became more pronounced when scene complexity increased (see Fig. 4). This effect did not reach statistical significance at the group level, but a closer look into individual data sets showed that the increase in scene complexity affected visual exploration behavior in four out of nine (44%) recovered patients. One of these patients neglected their contralesional field across all conditions and shifted their field of exploration even further towards the ipsilesional side when objects (medium) and dynamics (high complexity) were added to the scene (see Suppl. Material 4, Fig. 1, Patient 62). Three patients explored both sides of space in the low complexity condition equally and started to orient more towards the ipsilesional side when objects were added to the scene (medium complexity) (see Suppl. Material 4, Fig. 1, Patients 28, 32 and 36). In two patients (Patients 32 and 36), this ipsilesional orientation shift became even more pronounced in the high complexity condition when dynamics (movements and sounds) were added to the scene. Only one patient (Patient 28) showed the strongest ipsilesional orientation bias in the medium and then shifted their gaze back to the center of the visual field in the high complexity condition. Taken together, our results are in line with the predictions of the load-theory (Lavie, 1995, 2005; Lavie et al., 2014) as well as with previous study results on the effect of attentional load on unilateral neglect (Blini et al., 2016; Bonato et al. 2010, 2013; Deouell et al., 2005; List et al., 2008; Ogourtsova et al., 2018). However, some of the recovered patients did not show any orientation bias during free visual exploration irrespective of scene complexity. This finding has implications for our understanding of the recovery process as it suggests that the observed recovery from unilateral neglect might rely either on the recovery of primary function, namely spatial awareness, or on the acquisition of compensatory strategies.

**Fig. 2:**
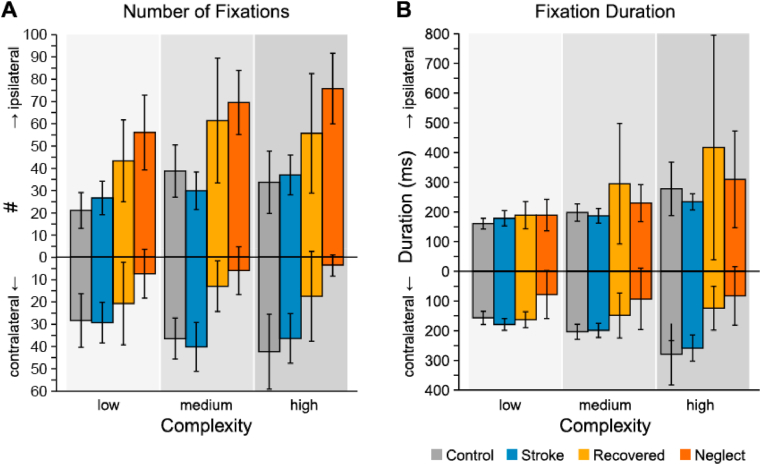
Average number of fixations (**A**) and fixation durations (**B**) on the ipsi- and the contralesional side for each group and scene complexity in ipsi- (upper bars) and contralateral direction (bottom). For control participants, fixations within the left visual field were classified as contralateral, as this was the case for the majority of patients as well. Error bars represent 95% confidence intervals.

**Fig. 3:**
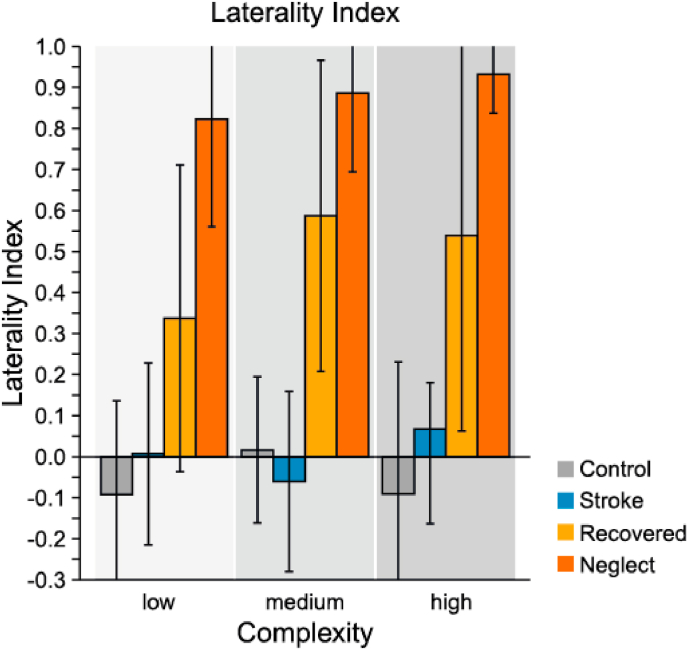
Laterality index (LI) for each group and scene complexity. LIs were calculated as the difference between number of fixations in ipsi- and contralesional direction divided by their sum. Values around zero indicate a balanced exploration behavior. For control participants, fixations within the left visual field were classified as contralateral, as this was the case for the majority of patients as well. Error bars represent 95% confidence intervals.

**Fig. 4:**
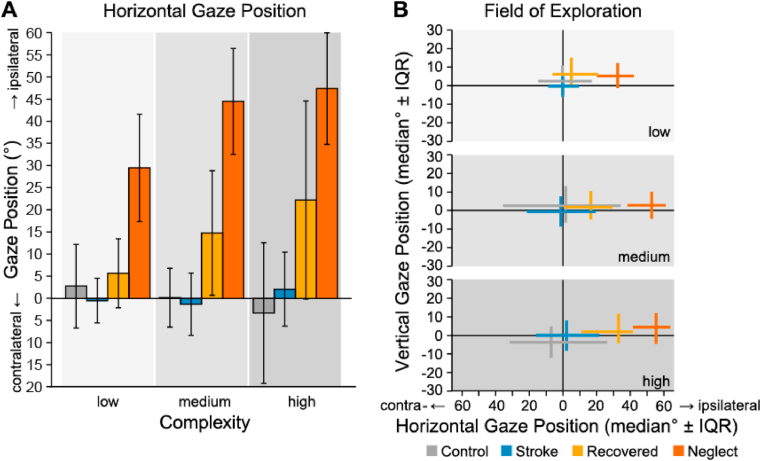
Spatial distribution of fixations. **(A)** Mean horizontal gaze position during fixations. Values around zero indicate a balanced exploration behavior. (**B**) Spatial distribution of the field of exploration quantified by median horizontal (vertical) gaze position, and horizontal (vertical) interquartile range. Note that the x-axis is coded as contra- (left) and ipsilateral (right), while the y-axis was not recoded. Values around zero indicate a balanced exploration behavior, and a larger interquartile range indicates a larger exploration field. For control participants, fixations within the left visual field were classified as contralateral, as this was the case for the majority of patients as well. Error bars represent 95% confidence intervals.

**Fig. 5:**
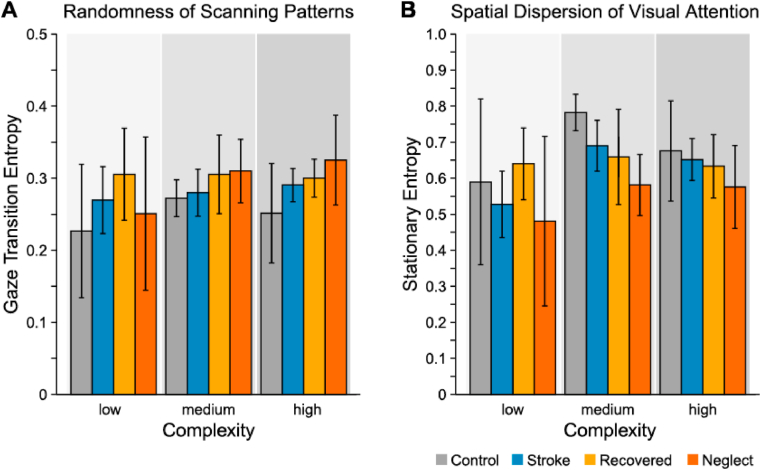
Visual scanning efficiency. **(A)** Mean gaze transition entropy as a measure of randomness of scanning patterns. Higher values indicate more random scanning paths and more frequent switching between viewed areas. (**B**) Stationary entropy as a measure of spatial dispersion of visual attention. Higher values indicate a wider distribution of fixations across the visual field. Error bars represent 95% confidence intervals.

**Fig. 6:**
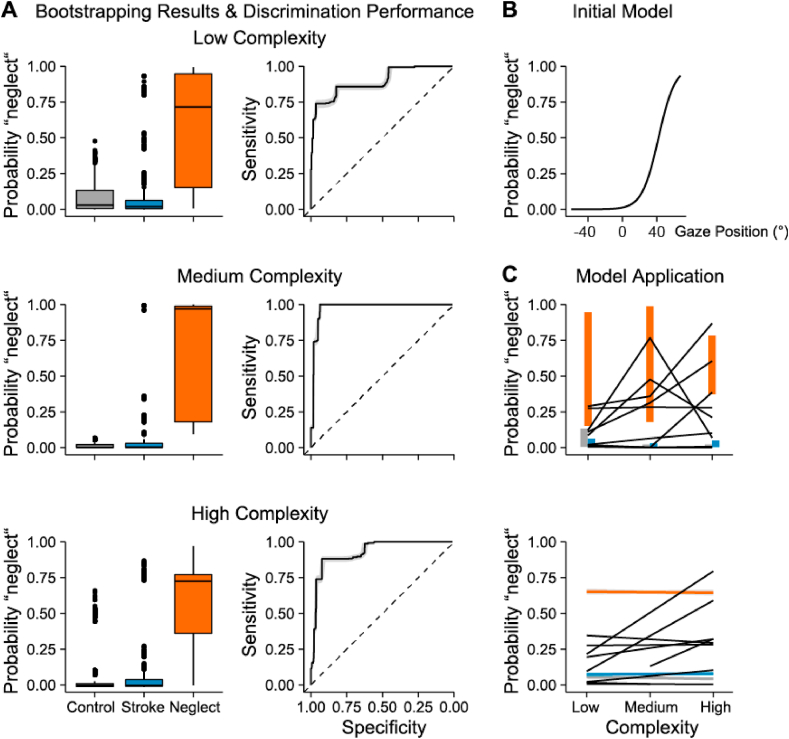
Summary of the prediction model results. **A)** Left: Boxplots of the outcome of the bootstrapping validation based on 1000 iterations. Right: ROC curves based on bootstrapping outcomes. Shaded error bars represent 95% CIs. **B)** Visualization of the initial model trained on control participants, stroke and neglect patients in the high complexity condition. Diverging curves demonstrate that a biased horizontal gaze position (towards the right on the x-axis) increases the probability of the diagnosis ‘unilateral neglect’. **C)** Top: Application of the prediction model on the group of apparently recovered neglect patients. Each line represents one of the 10 apparently recovered neglect patients. Colored bars represent the IQR based on bootstrapping results as depicted in panel A. Bottom: Regression analysis based on datapoints depicted in the left panel. Shaded error bars represent 95% CIs.

**Fig. 7:**
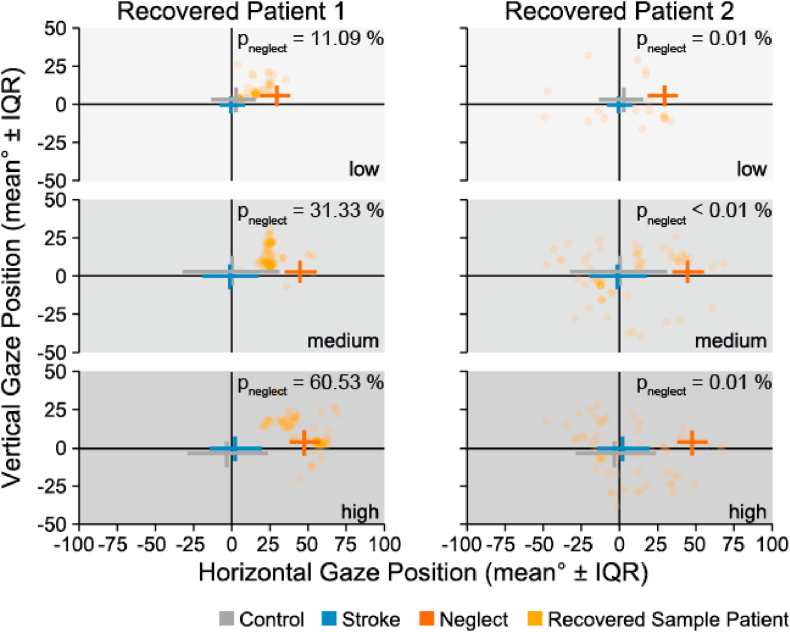
Visual exploration behavior of two recovered patients in comparison to the average of controls, stroke patients with no history of unilateral neglect and unilateral neglect patients. Single subject data of two patients belonging to the group of recovered neglect patients are shown as single fixations (i.e., each yellow dot represents one fixation). Recovered patient 1 (left) is a showcase of a recovered patient who still shows a pronounced preference for their ipsilesional and a consequent neglect of their contralesional side, especially in high complexity scenes, while recovered patient 2 has an even spatial distribution of fixations. Consequently, the model predicted an increasing likelihood of the diagnosis ‘neglect’ for patient 1, but not for patient 2. (For interpretation of the references to colour in this figure legend, the reader is referred to the Web version of this article.)

Furthermore, we found that healthy controls as well as stroke patients with no history of unilateral neglect widened their field of visual exploration when scene complexity increased. This effect was not shown in neither unilateral neglect nor recovered patients. This finding is in line with an earlier study, in which unilateral neglect patients did not widen their field of exploration when dynamic videos were shown instead of photos (Machner et al., 2012).

We trained a set of generalized linear models with different parameters of visual exploration behavior as potential predictors of unilateral neglect. We used eye tracking data of healthy controls, unilateral neglect patients and stroke patients with no history of unilateral neglect in order to identify the parameter which would robustly differentiate between the groups and be a suitable predictor of pathological visual exploration behavior. The best predictor was the mean horizontal gaze position in reference to the lesion site (*mean*, see Methods 2.6.3.). When estimating the probability of a unilateral neglect diagnosis for each individual recovered patient in each complexity condition, we found that three out of nine (33%) patients had an increased probability (P_neglect_ > 50%; *above chance*) of a unilateral neglect diagnosis when the scene complexity increased. We included graphic depictions of the spatial distribution of gaze of all recovered patients as well as their estimated probability of a unilateral neglect diagnosis in the supplementary material for better understanding (Fig. S1). The graphic depiction of each individual's explored visual field shows that the model estimated the probability of a unilateral neglect diagnosis rather conservatively, in that the orientation shift towards the ipsilesional side had to be extreme (mean > 40°) in order for the model to predict an increased probability (P_neglect_ > 50%) of unilateral neglect. Thus, even with a rather conservative criterion, the analysis of free visual exploration behavior revealed more patients with residual unilateral neglect than conventional tests. However, the model was based on a rather small sample and the estimated predictive values might change when more data are considered. Future studies with larger sample sizes and more changes in scene complexity might help to refine the model, with which not only the presence or absence of unilateral neglect could be estimated, but also symptom severity.

### 4.1 Limitations and recommendations for future studies

Our study results show that it is generally possible to differentiate between different degrees of unilateral neglect severity based on the analysis of eye movement patterns during free visual exploration. However, in order to create a diagnostic scale, it would be necessary to include more scenes with varying degrees of complexity, which should be presented in a random manner and validated in a larger sample size with both healthy participants of different ages and unilateral neglect patients. Moreover, scene complexity should be quantified and in order to control for asymmetries in the virtual scenes, the scenes should be flipped and a saliency analysis could be performed.

Another potential limitation of our study concerns the sample size and composition. As already mentioned, a larger sample size would help to refine the generalized linear model. Moreover, we included 17 left-hemispheric stroke patients, two of which were classified as recovered and 15 as “stroke patients with no history of unilateral neglect”. Thus, none of the left-hemispheric stroke patients was classified as suffering from unilateral neglect, which could potentially affect the validity of the model's predictions for these patients. Unilateral neglect is more common after right-sided strokes, but also occurs after left-hemispheric brain damage (Beume et al., 2017; Ringman et al., 2004). Right-sided unilateral neglect can occur both after right- and left-hemispheric strokes and although it has been shown that spatial attention deficits in right-sided unilateral neglect as measured by conventional tests seem to be less severe than in left-sided unilateral neglect, impairments affecting physical functioning and independence are similar (Ten Brink et al., 2017; Yoshida et al., 2022). Thus, every diagnostic tool for unilateral neglect should be validated for both right- and left-sided neglect. A previous study has already shown that eye movement analysis during free visual exploration is suitable to detect right-sided unilateral neglect after left-sided strokes and is even more sensitive to spatial attention deficits than cancellation tests (Kaufmann et al., 2021). However, as it has been shown that right-sided unilateral neglect is associated with different neuroanatomical correlates than left-sided unilateral neglect (Moore et al., 2021), future studies should separately analyze the effect of scene complexity on right-versus left-hemispheric stroke patients.

One major limitation for the clinical relevance of our study results concerns the availability of virtual reality and technical knowledge in the clinical setting. However, virtual reality is becoming increasingly popular in neurorehabilitation, which has already resulted in the development of more accessible and portable devices (Schiza et al., 2019), which could potentially make this technology more widely available in the clinical context.

### 4.2 Conclusion, implications and outlook

Our study results confirm that eye tracking is more sensitive to unilateral neglect than conventional tests (Cox and Aimola Davies, 2020) and that the mean horizontal gaze position during free exploration is a sensitive diagnostic marker of unilateral neglect (Kaufmann et al., 2020). We found that using the mean horizontal gaze position in reference to the lesion site as a diagnostic predictor allowed to sensitively differentiate between unilateral neglect patients, healthy controls and stroke patients with no history of unilateral neglect. Moreover, by increasing the complexity of the visual scene, we were able to identify three out of nine (33%) apparently recovered patients with an increased probability (P_neglect_ > 50%) of a unilateral neglect diagnosis as estimated by our generalized linear model using the mean horizontal gaze position as a predictor.

We systematically examined the impact of scene complexity on free visual exploration behavior of unilateral neglect patients in immersive virtual reality. This study provides the proof of concept for the manipulation of scene complexity during free exploration as one way to sensitively detect residual unilateral neglect and to differentiate between different degrees of symptom severity.

## Credit author statement

Kira Knoppe: Conceptualization, Methodology, Investigation, Writing – original draft. Nadine Schlichting: Formal analysis, Visualization, Writing – Review & Editing. Tobias Schmidt-Wilcke: Resources, Project administration, Writing – Review & Editing. Eckart Zimmermann: Data Curation, Funding acquisition, Software, Project Administration, Supervision, Writing- Review & Editing.

## Data Availability

I have shared the data at the Attach File step.
