# Supplementary material for: Increased scene complexity during free visual exploration reveals residual unilateral neglect in recovered stroke patients
Source: Neuropsychologia. 2022 Dec 15;177:108400. doi: 10.1016/j.neuropsychologia.2022.108400 (PMC9760574; doi:10.1016/j.neuropsychologia.2022.108400)
Supplement: Multimedia component 2 [file mmc2.docx]

# Supplementary Material

## Demographics and test results

**Table 1:** **Demographic characteristics and neuropsychological test results of unilateral neglect patients**. Patient 43 did not complete the neuropsychological tests because of extreme dizziness on testing day, but their severe unilateral neglect was confirmed by treating neuropsychologist.

| Subject | Age | Days  Since  stroke | Stroke  characteristics | | Lesioned hemisphere | Star  Cancellation | Line  Crossing | Baking Tray | Reading |
| --- | --- | --- | --- | --- | --- | --- | --- | --- | --- |
| 16 | 74 | 30 | ischaemic | right | | 0/15 | 0/16 | 4/8 | 27 |
| 20 | 68 | 37 | ischaemic | right | | 9/25 | 9/18 | 4/8 | 30 |
| 21 | 79 | 19 | ischaemic | right | | 18/22 | 17/18 | 4/8 | 30 |
| 43 | 79 | 26 | ischaemic | right | |  |  |  |  |
| 46 | 84 | 17 | ischaemic | right | | 1/7 | 0/11 | 0/12 | 25 |
| 61 | 89 | 28 | ischaemic | right | | 0/17 | 6/17 | 0/12 | 30 |
| 63 | 92 | 31 | ischaemic | right | | 0/14 | 0/3 | 0/12 | 30 |
| 38 | 73 | 36 | ischaemic | right | | 27/26 | 18/18 | 0/12 | 20 |

**Table 2: Demographic characteristics and neuropsychological test results of recovered patients**. These patients did not meet the criteria of a unilateral neglect diagnosis upon study admission, but suffered from unilateral neglect after their strokes as documented by their treating neuropsychologists.

| Subject | Age | Days  since  stroke | Stroke  characteristics | Lesioned  hemisphere | Star  Cancellation | Line  Crossing | Baking  Tray | Reading |
| --- | --- | --- | --- | --- | --- | --- | --- | --- |
| 19 | 86 | 20 | ischaemic | right | 26/27 | 18/18 | 6/6 | 30 |
| 28 | 75 | 15 | ischaemic | right | 22/22 | 18/18 | 6/6 | 30 |
| 32 | 75 | 35 | haemorrhagic | right | 24/25 | 18/18 | 6/6 | 30 |
| 36 | 86 | 28 | ischaemic | right | 26/25 | 18/18 | 6/6 | 30 |
| 39 | 83 | 23 | ischaemic | left | 27/27 | 18/18 | 6/6 | 30 |
| 42 | 68 | 25 | ischaemic | right | 20/24 | 18/18 | 6/6 | 30 |
| 60 | 63 | 21 | ischaemic | right | 24/26 | 18/18 | 6/6 | 30 |
| 62 | 85 | 57 | ischaemic | right | 26/27 | 18/18 | 6/6 | 30 |
| 44 | 72 | 119 | haemorrhagic | left | 27/27 | 18/18 | 6/6 | 30 |

**Table 3: Demographic characteristics and neuropsychological test results of stroke patients with no history of unilateral neglect.** Note: p48 and p50 did not complete the Line Crossing Task as both had troubles to correctly see the lines

| Subject | Age | Days since stroke | Stroke characteristics | Lesioned Hemisphere | Star Cancellation | Line Crossing | | Baking Tray | Reading |
| --- | --- | --- | --- | --- | --- | --- | --- | --- | --- |
| 2 | 70 | 24 | ischaemic | left | 27/27 | 18/18 | | 6/6 | 30 |
| 3 | 76 | 29 | ischaemic and haemorrhagic | right | 27/23 | 18/18 | | 6/6 | 30 |
| 4 | 84 | 20 | ischaemic and haemorrhagic | right | 27/27 | 18/18 | | 6/6 | 30 |
| 5 | 72 | 41 | haemorrhagic | left | 25/26 | 18/18 | | 6/6 | 30 |
| 6 | 64 | 30 | ischaemic | right | 23/27 | 18/18 | | 6/6 | 30 |
| 7 | 74 | 27 | ischaemic | left | 27/27 | 18/18 | | 8/4 | 30 |
| 8 | 76 | 32 | ischaemic | right | 27/26 | 18/18 | | 6/6 | 30 |
| 9 | 65 | 41 | ischaemic | right | 26/27 | 16/17 | | 6/6 | 30 |
| 10 | 74 | 31 | haemorrhagic | left | 27/27 | 18/18 | | 6/6 | 30 |
| 14 | 84 | 19 | ischaemic | right | 27/26 | 18/18 | | 6/6 | 30 |
| 15 | 65 | 27 | haemorrhagic | left | 27/27 | 18/18 | | 6/6 | 30 |
| 17 | 82 | 28 | ischaemic | left | 27/25 | 18/18 | | 6/6 | 30 |
| 23 | 72 | 20 | ischaemic | left | 27/27 | 18/18 | | 6/6 | 30 |
| 24 | 38 | 23 | ischaemic | left | 27/27 | 18/18 | | 6/6 | 30 |
| 25 | 83 | 27 | ischaemic | left | 27/27 | 18/18 | | 0/12 | 30 |
| 27 | 82 | 20 | ischaemic | right | 25/25 | 18/18 | | 6/6 | 30 |
| 33 | 81 | 14 | ischaemic | right | 23/26 | 18/18 | | 6/6 | 30 |
| 34 | 81 | 26 | ischaemic | left | 27/26 | 18/18 | | 6/6 | 30 |
| 37 | 76 | 32 | ischaemic | left | 21/25 | 18/18 | | 6/6 | 30 |
| 40 | 71 | 16 | ischaemic | left | 27/27 | 18/18 | | 6/6 | 30 |
| 41 | 88 | 61 | ischaemic | right | 24/27 | 18/18 | | 6/6 | 30 |
| 48 | 82 | 10 | ischaemic | right | 27/27 |  | 6/6 | | 30 |
| 49 | 68 | 41 | ischaemic | right | 23/26 | 18/18 | 6/6 | | 30 |
| 50 | 77 | 28 | haemorrhagic | right | 6/6 |  | 5/7 | | 30 |
| 51 | 66 | 21 | ischaemic | right | 27/24 | 18/18 | 6/6 | | 30 |
| 54 | 73 | 15 | ischaemic | right | 27/25 | 18/18 | | 4/8 | 30 |
| 55 | 68 | 31 | ischaemic | right | 27/27 | 18/18 | | 6/6 | 30 |
| 57 | 77 | 21 | ischaemic | left | 24/27 | 18/18 | | 6/6 | 30 |
| 59 | 80 | 30 | ischaemic | left | 27/27 | 18/18 | | 6/6 | 30 |

## Comparison between left- and right-sided stroke patients

In order to rule out that the differences in spatial orientation between stroke patients with no history of unilateral neglect, recovered patients and unilateral neglect patients were based on differences in lesion location, we compared right- and left-sided stroke patients with no history of unilateral neglect with regards to the spatial distribution of their fixations.

We computed three different two-way mixed ANOVAS analysing the impact of lesion location and scene complexity on the laterality index (LI) and the mean and median horizontal gaze position (median) of stroke patients with no history of unilateral neglect.

We did not find any significant main or interaction effects. Thus, differences between the groups (unilateral neglect, stroke without a history of unilateral neglect, recovered and controls) in the spatial distribution of fixations cannot be attributed to differences between right- and left-sided stroke patients.

## Comparisons between the experimental groups considering only right-sided strokes

### **Total number of fixations across all conditions**

A three-way mixed ANOVA analysing the impact of group, complexity and direction on the total number of fixations revealed main effects of complexity (*F*_(1.7,62.98)_ = 14.03, *p <* .001, η^2^*_p_* = 0.28), direction (*F*_(1,37)_ = 35.68, *p <* .001, η^2^*_p_* = 0.49) and interaction effects between group and direction (*F*_(3,37)_ = 12.96, *p <* .001, η^2^*_p_* = 0.51) and between complexity and direction (*F*_(5.11,71.54)_ = 3.78, *p <* .05, η^2^*_p_* = 0.09).

Pairwise post-hoc comparisons of the different complexity conditions did not reveal any significant differences between the conditions, thus, scene complexity did not alter the total number of fixations (all *ps >* .05). We found that more fixations were measured on the ipsi- than on the contralesional side across all conditions (*p <* .001). Decomposing the interaction effect between complexity and direction showed that the fixation asymmetry was not affected by changes in the visual content (all *ps >* .05). This effect occurred because of unilateral and recovered patients, who made significantly more fixations on their ipsilesional side across all conditions (both *ps <* .001). Controls and stroke patients with no history of unilateral neglect distributed their fixations evenly across both sides of space.

### Laterality index

A two-way mixed ANOVA analysing the impact of group and complexity on the laterality index revealed a main effect of group (*F*_(3,37)_ = 12.59, *p <* .001, η^2^*_p_* = 0.51).

Post-hoc pairwise comparisons of the groups revealed that unilateral neglect and recovered patients had significantly higher laterality indices than both controls and stroke patients with no history of unilateral neglect (all *ps <* .001), showing that they showed a significant imbalance in the spatial distribution of their fixations with more fixations on their ipsilesional side. Stroke patients with no history of unilateral neglect and controls did not differ from each other. Both groups had laterality indices of roughly zero, meaning that they evenly distributed their fixations on both sides of space.

### Interquartile range of horizontal fixation positions (IQR)

A two-way mixed ANOVA testing the effect of group and complexity on the interquartile range of horizontal fixation positions (IQR) showed significant main effects of group (*F*_(3,37)_ = 8.26, *p <* .001, η^2^*_p_* = 0.40), complexity (*F*_(2,74)_ = 4.92, *p <* .05, η^2^*_p_* = 0.12) and an interaction effect between group and complexity (*F*_(6,74)_ = 2.37, *p <* .05, η^2^*_p_* = 0.16).

Pairwise post-hoc comparisons between the groups revealed that controls had significantly higher IQRs than all other groups (all *ps <* .01), meaning that the field they visually explored was significantly wider than of all stroke patients. Pairwise post-hoc comparisons of the different scene complexities revealed that the visually explored space was significantly wider in the medium than in the low complexity condition (*p <* .01). This effect occurred only because healthy controls and stroke patients with no history of unilateral neglect widened their field of exploration when objects were added to the scene (both *ps <* .05). Scene complexity did not affect the width of the explored field in neither unilateral neglect nor recovered patients.

### Median horizontal gaze position

A two-way mixed ANOVA testing the impact of group and complexity on the median horizontal gaze position revealed significant main effects of group (*F*_(3,37)_ = 16.35, *p <* .001, η^2^*_p_* = 0.57), complexity (*F*_(1.65,61)_ = 9.62, *p <* .001, η^2^*_p_* = 0.21) and an interaction effect between group and complexity (*F*_(4.95,61)_ = 4.88, *p <* .001, η^2^*_p_* = 0.28).

Pairwise post-hoc comparisons of the groups revealed that unilateral neglect patients had significantly higher median gaze positions than all other groups (all *ps <* .05), indicating a stronger right-ward shift during exploration. Recovered patients oriented significantly less towards the right than unilateral neglect patients (*p <* .05), but more than controls and stroke patients with no history of unilateral neglect (both *ps <* .01). Both controls and stroke patients with no history of unilateral neglect had median horizontal gaze positions of roughly zero, indicating an even distribution of fixations across both sides of space. Thus, both unilateral neglect and recovered patients showed a strong right-ward (here: ipsilesional) orientation shift, which was more pronounced in the unilateral neglect group.

Pairwise post-hoc comparisons of the different complexity conditions did not reveal any significant difference (all *ps >* .05).

Decomposing the interaction effect of complexity and group revealed that the increase in scene complexity exacerbated the ipsilesional orientation shift in unilateral neglect patients (low vs. medium and high complexity, both *ps <* .05) and in recovered patients (low and medium vs. high, both *ps <* .05).

### Mean horizontal gaze position

Similar to the analysis of the median horizontal gaze position, a two-way mixed ANOVA testing the impact of group and complexity on the mean horizontal gaze position revealed significant main effects of group (*F*_(3,37)_ = 17.78, *p <* .001, η^2^*_p_* = 0.59), complexity (*F*_(1.56,57.7)_ = 8.43, *p <* .001, η^2^*_p_* = 0.19) and an interaction between group and complexity (*F*_(4.68,57.7)_ = 4.46, *p <* .001, η^2^*_p_* = 0.27).

Pairwise post-hoc comparisons of the groups showed that unilateral neglect patients oriented significantly more towards their right than all other groups (all *ps <* .01). Recovered patients showed a less extreme shift towards their right than unilateral neglect patients (*p <* .01), but also oriented more towards their right than controls and stroke patients with no history of unilateral neglect (both *ps <* .001). Stroke patients with no history of unilateral neglect and healthy controls did not differ significantly from each other as both groups had mean horizontal gaze positions of roughly zero, indicating an even spatial distribution of fixations across the whole width of the visual field.

Post-hoc pairwise comparisons of the different complexity conditions did not reveal significant differences between scene complexities (all *ps >* .05).

Decomposing the interaction effect between group and complexity revealed that recovered unilateral neglect patients had a significant higher mean horizontal gaze position in the high than in the low complexity condition (*p <* .01), meaning that they oriented significantly more towards the right (here: their ipsilesional side).

## Spatial distribution of gaze of individual recovered patients


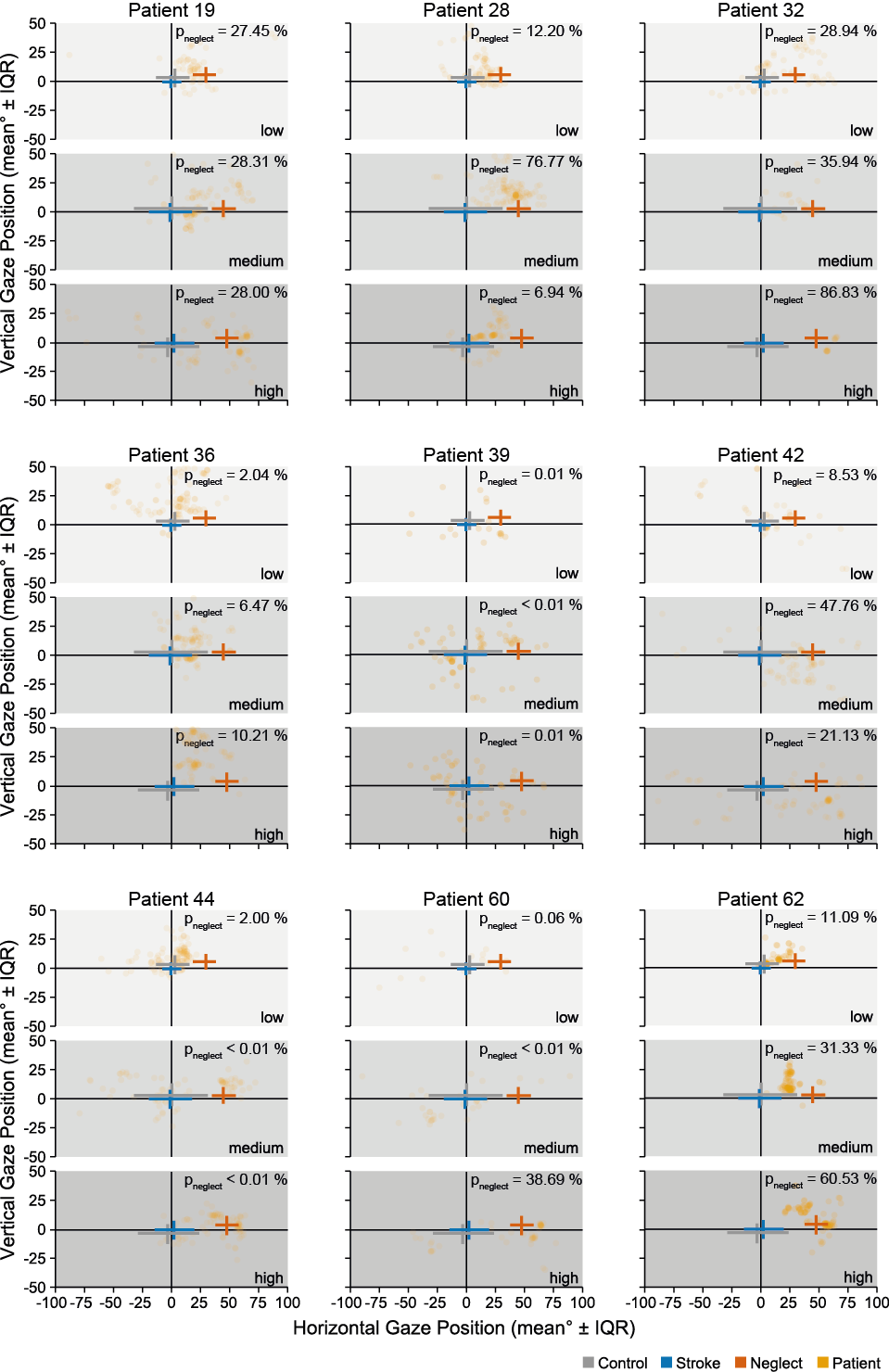


**Figure 1: Visual exploration behavior of all individual recovered patients** in comparison to the average of controls, stroke patients with no history of unilateral neglect and unilateral neglect patients. Single subject data of individual patients belonging to the group of recovered neglect patients are shown as single fixations (i.e., each yellow dot represents one fixation). For each patient, the likelihood of a ‘unilateral neglect’ diagnosis in each complexity condition is depicted in the outer right corner of each graph.
